# The Organization of Collective Group Movements in Wild Barbary Macaques (*Macaca sylvanus*): Social Structure Drives Processes of Group Coordination in Macaques

**DOI:** 10.1371/journal.pone.0067285

**Published:** 2013-06-21

**Authors:** Anne Seltmann, Bonaventura Majolo, Oliver Schülke, Julia Ostner

**Affiliations:** 1 Primate Social Evolution Group, Courant Research Centre Evolution of Social Behaviour, Georg-August University Göttingen, Kellnerweg, Germany; 2 School of Psychology, University of Lincoln, Brayford Pool, Lincoln, United Kingdom; 3 Courant Research Centre Evolution of Social Behaviour, Georg-August University Göttingen, Kellnerweg, Germany; Texas A&M University, United States of America

## Abstract

Social animals have to coordinate activities and collective movements to benefit from the advantages of group living. Animals in large groups maintain cohesion by self-organization processes whereas in smaller groups consensus decisions can be reached. Where consensus decisions are relevant leadership may emerge. Variation in the organization of collective movements has been linked to variation in female social tolerance among macaque species ranging from despotic to egalitarian. Here we investigated the processes underlying group movements in a wild macaque species characterized by a degree of social tolerance intermediate to previously studied congeneric species. We focused on processes before, during and after the departure of the first individual. To this end, we observed one group of wild Barbary macaques (*Macaca sylvanus*) in the Middle Atlas, Morocco using all-occurrence behaviour sampling of 199 collective movements. We found that initiators of a collective movement usually chose the direction in which more individuals displayed pre-departure behavior. Dominant individuals contributed to group movements more than subordinates, especially juveniles, measured as frequencies of successful initiations and pre-departure behaviour. Joining was determined by affiliative relationships and the number of individuals that already joined the movement (mimetism). Thus, in our study group partially shared consensus decisions mediated by selective mimetism seemed to be prevalent, overall supporting the suggestion that a species’ social style affects the organization of group movements. As only the most tolerant species show equally shared consensus decisions whereas in others the decision is partially shared with a bias to dominant individuals the type of consensus decisions seems to follow a stepwise relation. Joining order may also follow a stepwise, however opposite, relationship, because dominance only determined joining in highly despotic, but not in intermediate and tolerant species.

## Introduction

Living in groups has many advantages. Social animals avoid predators, collaborate to defend territories, attract mates and profit from each other’s knowledge about the habitat [1], [2]. In order to maintain the cohesion of a social group, individual group members need to synchronize their activities and coordinate their behaviour, especially when moving from one place to another, e.g. from feeding to sleeping areas [3]. The success of coordination, therefore, crucially influences individual fitness and selection should favor individuals who are able to move cohesively [3]. Important issues are the decisions about when and where to move but group members often differ in their preferred travel timing and destination due to individual differences in motivation, knowledge of the home range, physiological and morphological traits which leads to “consensus costs”, i.e. reduced fitness, when individuals give up their preferred activity to abide to the group’s decision [4], [5].

The majority of the few empirical studies on group coordination in non-human primates conducted in the last decade were carried out in semi-free ranging conditions with *ad libitum* access to food and in the absence of predators which may diminish true conflicts of interest [6]-[9], but see [10]-[13]. Thus, it remains to be investigated whether the same patterns can be found in the wild where failure to coordinate or continued compromises of the same individuals’ interests may lead to an actual cost for group members [12]. Therefore, studies on semi-free ranging animals are well-suited for the investigation of behavioural mechanisms [14] and they should be repeated under natural conditions. As definitions of group movement related terms differ pronouncedly between studies making comparisons among them difficult, it has been proposed to a priori define group movements per taxon or even per group [15] to account for confounding variables such as ecological conditions, group size, group composition and cohesion which all may affect home range size and travel distances [16]. The aim of this study thus was (i) to develop operational definitions of group movement related terms for this species following Pyritz et al. [14] and (ii) to investigate the processes underlying group coordination and decision-making in a group of wild Barbary macaques (*Macaca sylvanus*) to (iii) eventually allow comparisons with other species.

Group decision is defined as “a decision made by the animals within a group” where individuals can choose among two or more alternative behaviours [4], [17]. This can be met either by (1) equally shared consensus decisions, (2) partially shared consensus decisions or (3) unshared consensus decisions. Consensus decisions raise important questions about the influence of particular individuals, communication mechanisms, information gathering and the ability to cooperate within the group [11]. Individuals aim to reach a consensus and therefore make trade-offs [4], [11], e.g. followers need to abide to the leading animal(s) to maintain group cohesion [18]. In equally shared consensus decisions (1), all members contribute equally to the decision outcome, regardless of age, rank sex or other variables [15]. In partially shared consensus decisions (2) some group members (e.g. dominant individuals [8], [11]) have a greater influence on the movement decision [15]. An unshared consensus decision (3) is displayed if one particular individual decides on behalf of all other group members who all have to follow this decision [15].

In large groups, such as flocks of birds or shoals of fish, individual recognition is not self-evident and communication is only possible between spatial neighbours (“local communication”, [4]). In contrast, in small groups with individual recognition, every group member can usually communicate with others (“global communication”). The decision to move can be mediated by a) self-organization processes, i.e. the individual group members follow local behavioural rules which results in organized behaviour by the whole group without the need of a global control by certain individuals or b) consensus decisions [4], [15]. It is also possible that a joining process underlies both mechanisms [19]. One self-organization process is mimetism, i.e. the higher the number of individuals already performing a behaviour, e.g. joining a movement, the higher the probability that another individual will perform this behaviour as well, with quorum thresholds. A quorum is defined as “the minimum number, i.e. threshold, of group members that need to favor a particular action for the whole group to adopt this action” [4], [15]. Mimetism can be anonymous (allelomimetism), meaning that either the identity of the individuals is irrelevant for the decision process or that social relationships are either equally distributed across all possible dyads or do not affect the amplification process [5]. Alternatively, the decision to just mimic the behavior of a group mate may be selective and guided by the genetic or social relationship with other individual (“selective mimetism”, [19]). In shared consensus decisions, the consensus is usually determined by a quorum or by averaging across votes. Voting is defined as communicating a preference with regard to the decision outcome [15] and has been described during the pre-departure period.

The pre-departure period has a crucial impact on the following movement. It is defined as the “period preceding the departure of the initiator” and is “delineated by the presence of pre-departure behaviours” [15]. Its quality, intensity and simultaneity are thought to influence the decision-making process [20], [21]. Pre-departure behaviour is interpreted as “voting” or negotiating for a certain direction [22], [8], [9] and group members try to reach a quorum threshold for departure [15] via vocalizations (e.g. mountain gorillas, *Gorilla gorilla beringei* [23]) or stereotyped movements (e.g. Bewick’s swans, *Cygnus columbianus* [24]). Macaques were observed to display pre-departure behaviour in the form of moving away from the group and repeatedly glancing backwards [25], [26]. If various individuals participate in pre-departure behaviour it indicates that the final decision is shared between group members and that an unshared consensus is unlikely [8].

Once the group during the pre-departure period has decided on a specific time and direction, one individual has to initiate the collective movement. Some individuals are more likely to act as leaders due to their age, dominance status, sex, social integration, personality, dispersal status and therefore better knowledge about the environment or because of their nutritional needs being the highest among the group [27], [16], [13]. Since these variables may covary, it is difficult to identify the most important factor [16] and to disentangle their relative importance [28]. In two socially intolerant macaques species (Japanese, *M. fuscata* and rhesus macaque, *M. mulatta*) with a steep dominance gradient, old and dominant individuals were often in the front of group movements [7], [8], [12]. Males also have been shown to be at the front of group movements [29], [30], [8], [31], while in other studies females to led the group [32], [33], [13]. Other studies attributed the direction of the group to knowledgeable individuals [18]. Additionally, the precise progression of the remaining group members order may give insight to processes underlying consensus decisions [25].The joining behaviour may be crucially influenced by social relationships in species where individual recognition is possible [21] and affiliative relationships have been shown to mediate coordination in non-human primates [19], [26]. Furrer et al. [34] stressed that the role of initiators can be influenced by a variety of variables which may apply for the progression order was well. Therefore, comparative studies on species where some of these variables are constant would be ideal to make progress in this research area.

To tackle the problem of covarying variables, studies on species which have several traits in common but differ in others are ideal to make progress in the research of the organization of group coordination. The genus *Macaca* fulfills this criterion. The social structure of macaques differs among the 20 species [35], but all macaques form multi-male multi-female groups and are structured into matrilines of philopatric females [36]. Differences in social structure, namely social tolerance among females, have been linked to differences in e.g. play behaviour [37], [38], patterns of migration [39] and group decision-making [7], [8]. The lack of age and rank effects on the frequency of movement initiation and the lacking relation between social bonding patterns and the joining order observed in Tonkean macaques has been suggested to result from their high degree of social tolerance and the lack of kin preference in social behaviour. Japanese macaques mark the opposite end of the social tolerance spectrum among macaque females with highly nepotistic and intolerant relationships that are thought to explain the strong dominance and kin effects on the joining process in collective movements [12].

In one of the most tolerant, individualistic and egalitarian macaque species, equally shared consensus decisions with a progression order based on affiliative relationships were found, whereas in two of the most despotic, nepotistic and intolerant macaques, partially shared consensus decisions with joining according to kinship and dominance were found [7], [8], [12]. To further investigate this relationship between social structure and organization of collective movements, we provide data on a species with an intermediate degree of social tolerance among females [40], the Barbary macaque. In Tonkean and Barbary macaques, the steepness of the dominance hierarchy is moderate to low whereas it is high in Japanese and rhesus [41]. The degree to which females prefer kin for social interactions and coalitions is less pronounced in Barbary macaques than in Japanese and rhesus macaques [36]. The level of counter-aggression between nonkin is high in Tonkean (> 60%), intermediate in Barbary (about 40%) and low (< 10%) in Japanese and rhesus macaque females [42]. Barbary and Tonkean macaque females usually reconcile after conflicts, in contrast, the conciliatory tendency in Japanese and rhesus macaques is much less pronounced [42].

More precisely the aims of this study were (1) to elucidate the role of the pre-departure period on the direction of a collective movement, (2) to identify the type of group decision-making and the mechanism underlying the joining process and (3) to compare the findings to other macaque species. We predicted (1) a crucial effect of displaying pre-departure behaviour on the direction of a collective movement. Due to the hierarchical society of macaques we expected (2) partially shared consensus decisions where adult and dominant, socially integrated, individuals play a more prominent role than juveniles, subordinate or socially less integrated group members. This could be expressed in the higher frequency of initiation movements and/or the initiation success of these individuals. As macaques are highly social and form strong bonds within the group we predicted an effect of the affiliative relationships on the progression order during collective movements, e.g. dyads with a strong bond should travel together. Finally, we predicted the joining process also to be influenced by mimetism and to find adult males at the front of progression orders similar to other species with male dominance.

## Methods

### Ethics statement

This study complies with Moroccan, German and UK regulations regarding the ethical treatment of research subjects. Permission to conduct the study was given by the Haut Commissariat des Eaux et Forêts and the Lutte Contre la Désertification, Morocco (no permission IDs were given). The study was fully observational and our data collection did not affect the monkeys' welfare.

### Study site and subjects

The study was conducted at the Ifrane National Park near the town Azrou in the Middle Atlas Mountains of Morocco (33°24′9N - 005°12′9W) at an altitude of about 1730 to 1930 m a.s.l. The terrain and forest environment consists of open mixed cedar and oak woodland with undergrowth which provides high visibility. A road crosses through the home range and occasionally, shepherds with sheep and dogs pass by near the edge of the home range. A wild, well-habituated Barbary macaque group was observed by two observers from March to June 2012. The group consisted of six adult females, six adult males, one sub-adult male (4 yrs.), nine juveniles and six infants that were born during the study period (Table 1). For all analyses the single sub-adult male was included in the adult age class. Apart from the births, the group composition was stable during the study period and every individual was present on every observation day. Study subjects were all group members except the infants. All individuals were recognized by natural markings like moles, scars and fur color pattern.

**Table 1:** Composition of the study group as well as information on age class, ordinal rank, and social integration.

ID	Sex	Age	Rank	CIS	Code
Oz	m	adult*	1	0.86	M1
Ar	m	adult*	2	0.96	M2
Lw	m	adult*	3	0.77	M3
Ge	m	adult*	4	0.39	M4
Si	m	6 years	5	0.79	M5
Nd	m	adult*	6	0.5	M6
An	f	adult*	7	1.25	F1
Mc	m	4 years	8	0.87	SM1
Da	f	adult*	9	1.46	F2
Jo	f	adult*	10	0.71	F3
Ke	f	adult*	11	0.81	F4
Rb	f	adult*	12	0.96	F5
He	f	adult*	13	0.72	F6
Dk	f	3 years	14	1.28	JF1
Aj	m	2 years	16	1.47	JM1
Kr	f	3 years	16	0.98	JF2
Rf	m	2 years	16	1.34	JM2
Kl	m	2 years	18	1.21	JM3
Ak	m	1 year	19	1.51	JM4
Dn	f	2 years	20	1.47	JF3
Jj	m	1 year	21	1.16	JM5
Do	f	1 year	22	1.34	JF4
Hs	m	1 year	23	1.20	JM6
Rl	f	1 year	24	1.23	JF5

m  =  male, f  =  female, *individuals were already fully grown at the beginning of habituation of the group three years ago, exact age is therefore not known, CIS  =  composite index of social integration, see methods for details.

### Definitions

We decided to not force a priori definitions for what a movement is compared to any locomotor activity occurring during regular activities like feeding and foraging. Instead, we conducted a two week pilot study where we collected data on the distance covered and latencies between individuals’ bouts of locomotion and derived the following definitions according to the procedure suggested by Pyritz et al. [14]. Thus, we defined group movement related terms specifically for this social group of Barbary macaques in their specific habitat (Fig. S1):

- *Initiator:* The individual moves a minimum distance of 18 meters in a directed manner as straight as environmental conditions allow (bypassing natural obstacles, such as deep valleys was still included in the definition of straight forward) without pausing for more than two seconds. Movements within a social context, e.g. chasing or approaching another individual, or as a response to alarm calls were excluded. To qualify as an initiation movement, at least three individuals needed to be more than 11 meters behind the initiator.
- *Termination:* The initiation movement ended, when the initiator was stationary again for at least 3.5 minutes.
- *Followers:* Group members moving behind the initiator were called followers unless their movements diverged more than 45° from the initiator’s trajectory. Otherwise, the direction was considered as different and the individual may have been an initiator of a different movement. Followers had to arrive within an 11 meter-radius around the terminator, no later than 3.5 minutes after the termination of the movement. Animals that were already there when the initiator terminated the movement were not considered followers.
- *Successful movement:* A movement was successful if the initiator had three followers [6], [15].

### Behavioural observations

All occurrences sampling [43] was used to investigate the type and underlying mechanisms of decision-making before and once an individual initiated a movement. Two observers recorded simultaneously the identity of group members conducting pre-departure behaviour (incentive movements or back glances, [9], [26]) one observer focusing on the front, the other at the main part of the group. An incentive movement was defined as a directed walk of an animal for a distance shorter than to be accounted for as an initiation movement which does not result within 2 seconds in feeding, social interactions, lying down or climbing a tree. A back glance was defined as a turn of an individual’s head of more than 90°. Back glances during feeding or social interactions were not considered to be relevant for pre-departure behaviour and therefore excluded. If the directions of pre-departure behaviours formed an angle exceeding 45° the directions were considered to be different [9], [26].

Once one individual initiated a group movement, one observer focused on the initiator and recorded the identity of the initiator, the time of its departure and the identity of followers. The second observer recorded the exact progression order of the joining individuals and the time of their departure. A joiner was defined as an individual moving in an angle of less than 45° to the initiators trajectory and crossing an imaginary line situated 6 meters (a third of the minimum distance one individual had to move to initiate a group movement) behind the initiators start point within 10 minutes. If the initiator started in the centre of the group and individuals ahead of it walked at least 6 meters in an angle of less than 45° to the initiators trajectory, they were counted as joiners as well. When group movements were disrupted by dogs, their barks or cars when crossing the road, only the progression order until the disruption but no other parameters were recorded to exclude external influences. When the initiator returned to the group, the observation was cancelled.

To gain information about the social centrality of individuals and affiliative relationships between group members, instantaneous scan sampling [43] was conducted every hour outside a moving context. We recorded for each group member all individuals (1) in body contact, (2) the number of individuals within 1.5 meters and (3) within 5 meters, whereas all surrounding individuals were only accounted once, in the closest possible category. Study subjects who were not found after 15 minutes were discarded from the scan. If an initiation happened during the scan, the scan was cancelled. Only scans with more than 80% of group members present (*N* = 122) were used in the analysis.

Information about dominance relations between individuals was acquired via *ad libitum* recording of agonistic behaviour (aggression: lunge, charge, chase, slap, grab, jump on, bite, ground slap, run towards, open mouth, head bob; submission: make room, give ground, flee, crouch, present submission, fear scream, [44], [45]). An interaction was rated as decided, if only two monkeys were involved in a conflict, maximum one showed aggressive behaviour and only one acted submissively. In total, 850 decided conflicts were recorded.

### Statistical Analyses

All statistical tests were conducted in R-2.14.1 (R Development Core Team (2010) R: a language and environment for statistical computing. R Foundation for Statistical Computing, Vienna). Tests were two-tailed and the significance level was set to α = 0.05. If not reported otherwise, means and standard errors are given in the format X ± SEM. Correlations were computed via Spearman’s rank correlation. A Paired Mann-Whitney *U* test was conducted to compare the number of individuals that showed pre-departure behaviour for the eventually chosen and unchosen direction of the initiator. If an individual changed the direction of its pre-departure behaviour the most recent one was taken into account and the preceding ones discarded. The data on the progression order were used to calculate a travel association score as follows:
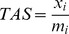



Where x_i_ is the number of group movements where both animal A and B directly travel together and m_i_ is the mean of group movements where two animals travel together for all dyads. If two or more individuals crossed the progression line simultaneously they were all rated associated with each other and additionally with the preceding and subsequent individual. The elapsed time between two joining individuals was defined as departure latency [19]. If one individual crossed the progression line several times, the departure latencies of this progression order was excluded from data analyses from this point on. Only progression orders with 15 and more individuals (> 62% of the group) were analyzed to build the travel association scores and to determine departure latencies.

A Chi-squared test was conducted to test whether every individual had the same probability to be in the scans (Chi-squared test: *χ^2^* = 1.59, *df* = 23, *P* = 1). A dyadic index of social relationships (DIS) was calculated by adding the number of scans where two individuals were seen in body contact (grooming or resting) divided by the average of this number for all dyads of the group similar to Silk et al. [46]. High values indicate a stronger than average bond within a dyad. By combining the three parameters (1) how many individuals are in body contact, (2) within 1.5 meters and (3) at a distance between 1.5 and 5 meters, we acquired an estimator of the centrality of individuals, the composite index of social integration (CIS). This was done similar to Sapolsky et al. [47] and Silk et al. [48] as follows:
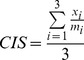



Where x_i_ was the value for individuals in (1) body contact, (2) at a distance up to 1.5 meters, (3) between 1.5 and 5 meters and m_i_ is the median values for (1), (2) and (3). The dominance hierarchy was constructed by calculating the normalized David’s Score via the win proportions P_ij_ [49] and was found to be linear (MatMan, males: Landau Index h’ = 0.93, females: h’ = 1, juveniles: h’ = 0.99; [50]).

## Results

The process of a collective movement can be divided into several steps: (i) pre-departure period, (ii) initiation movement of an individual, (iii) joining by other group members and (iv) following behaviour of individuals to a new destination. Below, each step will be addressed separately.

### Pre-departure period

**Figure 1:**
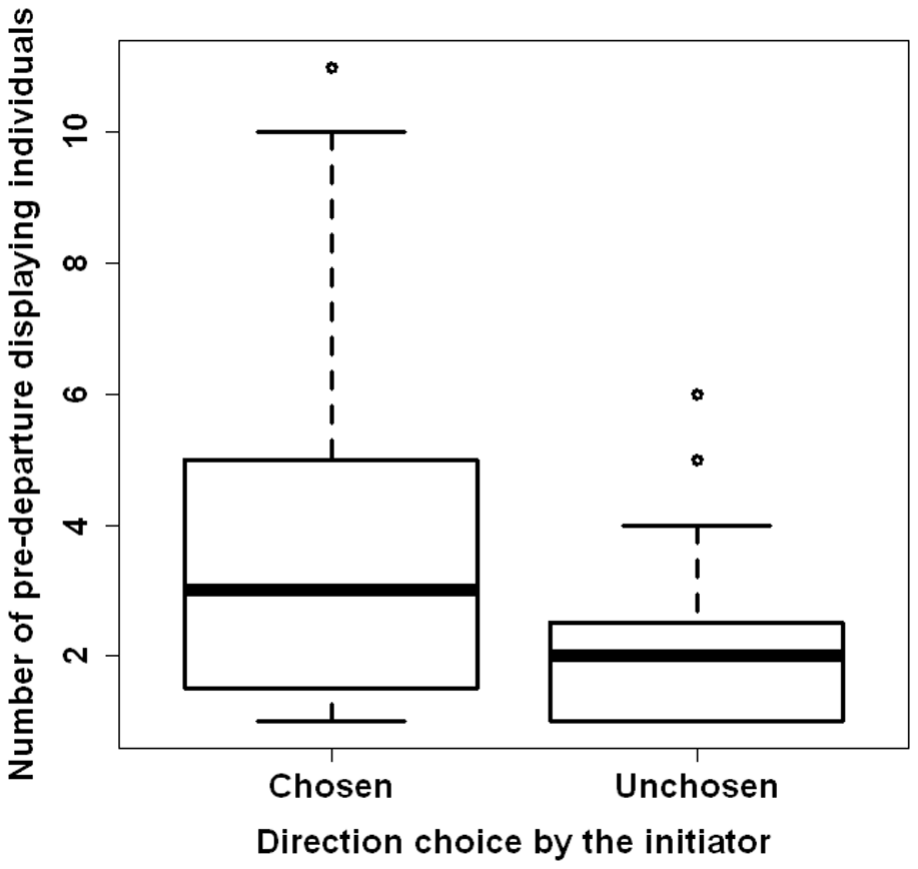
Number of pre-departure displaying individuals in the eventually un-/chosen direction of the initiation movement. The direction of the following successful initiation movement had more individuals “voting” for this than the unchosen direction during the pre-departure period (Paired Mann-Whitney *U* test: *V* = 374, *N* = 36, *P*<0.05). The figure shows medians (bold line), 25 – 75% percentiles (box), 5 – 95% (percentiles whiskers), and outliers (points).

In total, 352 initiation movements were observed. Of these 199 (56.53%) were successful. From the successful initiation movements 156 (78.39%) were preceded by pre-departure behaviour by at least one individual (range: 1 – 12, mean  = 2.78±2.49 individuals). Prior to 104 of 199 (52.26%) successful initiation movements, the initiator displayed pre-departure behaviour. In 26, i.e. in 13.1% of collective movements, it was the first individual to show pre-departure behaviour. If pre-departure behaviour was displayed for different directions, the direction of the following successful initiation movement had more individuals “voting” for this direction than for the other one (successful direction: 1 – 11, mean: 3.44±2.59 individuals, unsuccessful direction: 1 – 6, mean: 2.08 ± 1.40 individuals; Paired Mann-Whitney *U* test: *V* = 374, *N_direction1_* = *N_direction2_* = 36, *P*<0.05, Fig. 1). We never observed the initiator changing the direction of its pre-departure behaviour.

### Initiatorship

**Figure 2:**
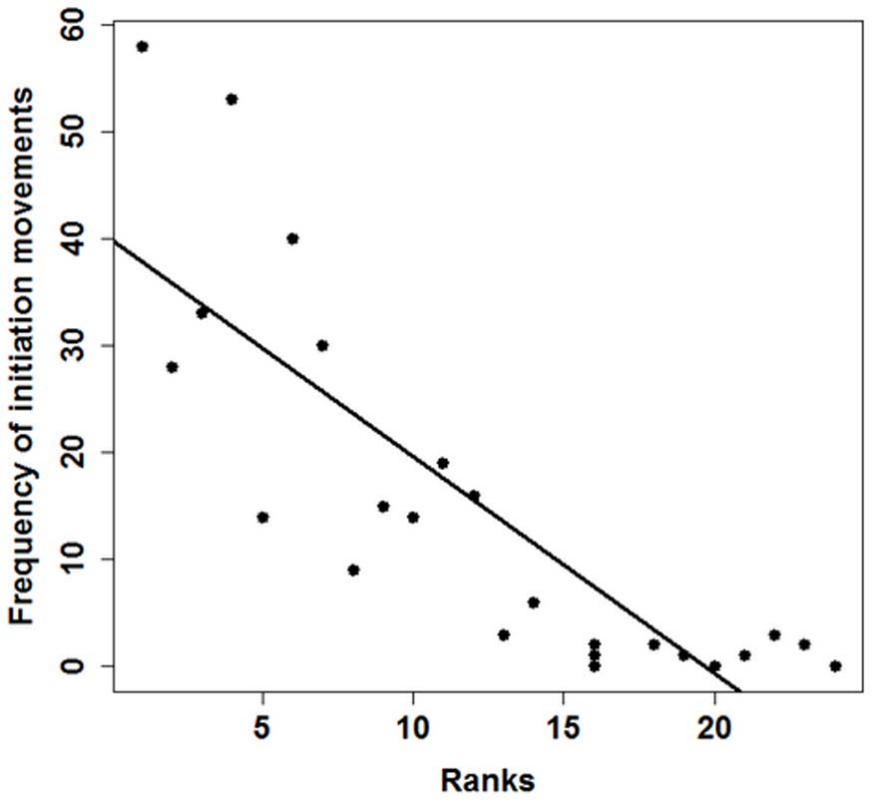
Influence of the hierarchical rank order on the frequency of attempted initiation movements. Apart from 2 of 13 adults (rank 8 and 13), every adult group member tried to initiate at least 14 collective movements. Juveniles (rank 14 – 24) tried to initiate 0 – 6 collective movements. The higher ranking an individual the more often it tried to initiate a group movement (Spearman rank correlation: *ρ* = –0.89, *N* = 24, *P*<0.0001).

The highest ranking male M1 tried to initiate 58 collective movements. Three juveniles (2 year old male, 2 year old female, 1 one year old female ranking 16, 20 and 24, respectively) never tried to initiate a group movement. The higher ranking an individual the more often it tried to initiate a group movement, regardless of whether juveniles were included in the test or not (Spearman rank correlation: all individuals: *ρ* = -0.89, *N* = 24, *P*<0.0001; only adults: *ρ* = -0.69, *N* = 13, *P*<0.01, Fig. 2). Since all females were lower ranking than adult males, adult males tried to initiate group movements more often than females.

When considering all group members, individuals with a lower CIS tried to initiate collective movements more frequently than more central individuals (Spearman rank correlation: *ρ* = -0.63, *N* = 24, *P*<0.0001). When juveniles were excluded, the frequency of initiation movements did not depend on the CIS (Spearman rank correlation: *ρ* = -0.15, *N* = 13, *P* = 0.63). This may be due to the fact that rank and CIS were significantly, positively correlated, but also only when considering all individuals (Spearman rank correlation: *ρ* = 0.59, *N* = 24, *P*<0.01). When juveniles were not considered, this effect disappeared as well (Spearman rank correlation: *ρ* = 0.06, *N* = 13, *P* = 0.84).

**Figure 3:**
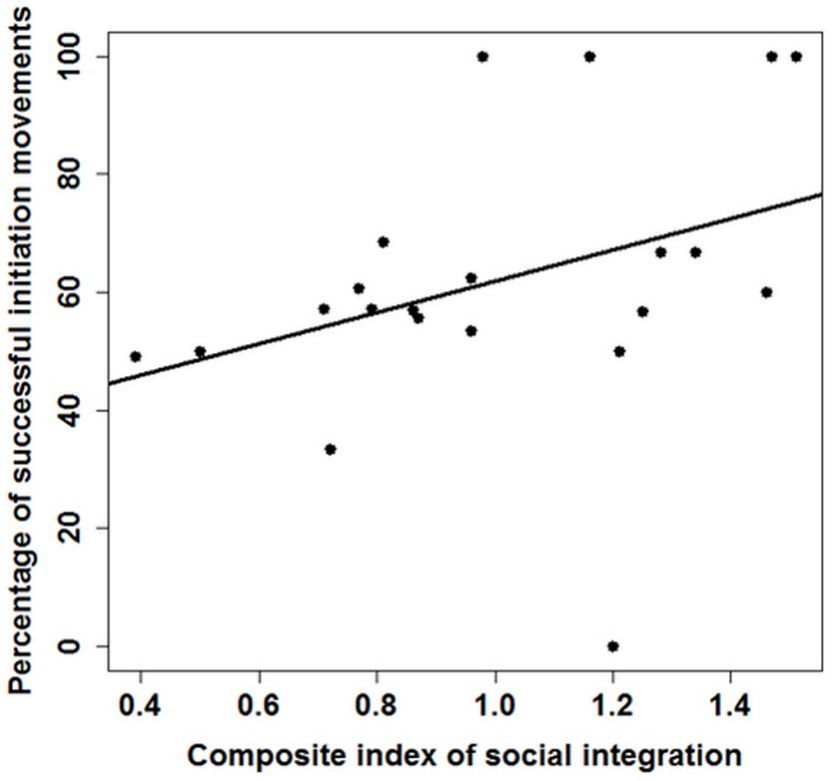
Influence of social integration on the percentage of successful initiation movements. The higher the CIS (composite index of social integration) of an individual the higher the percentage of successful initiation movements (Spearman rank correlation: *ρ* = 0.48, *N* = 21, *P* > 0.05).

When considering all initiation attempts, the percentage of successful initiation attempts by individual initiators was not rank dependent, even when excluding juveniles (Spearman rank correlation: *ρ* = 0.35, *N* = 21, *P* = 0.12, adults only: Spearman rank correlation: *ρ* = 0.20, *N* = 13, *P* = 0.51). The percentage of successful initiation movements of all initiation attempts by the individual initiators was dependent on the social centrality of group members: individuals with a higher CIS had a higher percentage of successful initiation movements than those with a lower CIS (Spearman rank correlation: *ρ* = 0.48, *N* = 21, *P* > 0.05, Fig. 3). This effect disappeared when excluding juvenile group members (Spearman rank correlation: *ρ* = 0.38, *N* = 13, *P* = 0.20).

Collective movements initiated by juveniles were of shorter distance than those by adults and occurred rarely (juveniles: 55 ± 31 meters, *N* = 12; adults: 106 ± 123 meters, *N* = 183). The distance of a collective movement depended on rank. The higher ranking an individual, the larger the average distance covered during a successful initiation movement (Spearman rank correlation: *ρ* = –0.66, *N* = 20, *P*<0.01). The CIS did not affect the distance of collective movements (Spearman rank correlation: *ρ* = –0.16, *N* = 20, *P* = 0.51). Results did not change when excluding juveniles (Spearman rank correlation, rank: *ρ* = –0.59, *N* = 13, *P*<0.05; CIS: *ρ* = 0.16, *N* = 13, *P* = 0.60).

**Figure 4:**
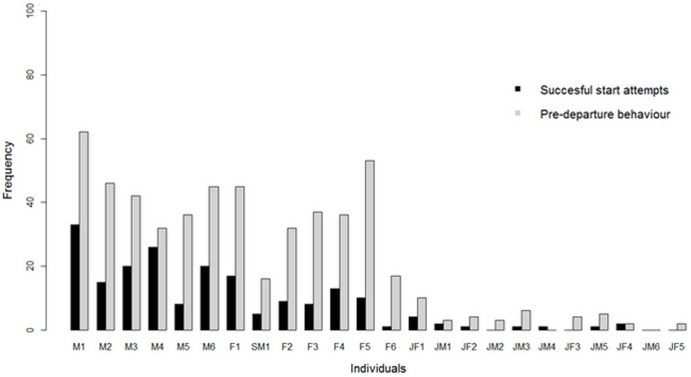
Frequency of contribution to group movements by individual. Individuals are arranged in order of decreasing dominance from left to right.

The individual contribution to the number of successful initiation movements and the number of displaying pre-departure behaviour prior to group movements varied with rank (Fig. 4). The higher ranking an individual, the higher was the number of successful start attempts it achieved (Spearman rank correlation: *ρ* = –0.89, *N* = 24, *P*<0.001), regardless of the number of unsuccessful start attempts. This effect remained significant even when excluding juveniles from the analysis (Spearman rank correlation: *ρ* = -0.70, *N* = 13, *P*<0.01). The higher ranking an individual, the more often it showed pre-departure behaviour prior to collective movements (Spearman rank correlation: *ρ* = –0.86, *N* = 24, *P*<0.001), but this effect disappeared when excluding juveniles from the analyses (Spearman rank correlation: *ρ* = –0.40, *N* = 13, *P* = 0.17).

### Joining process

**Figure 5:**
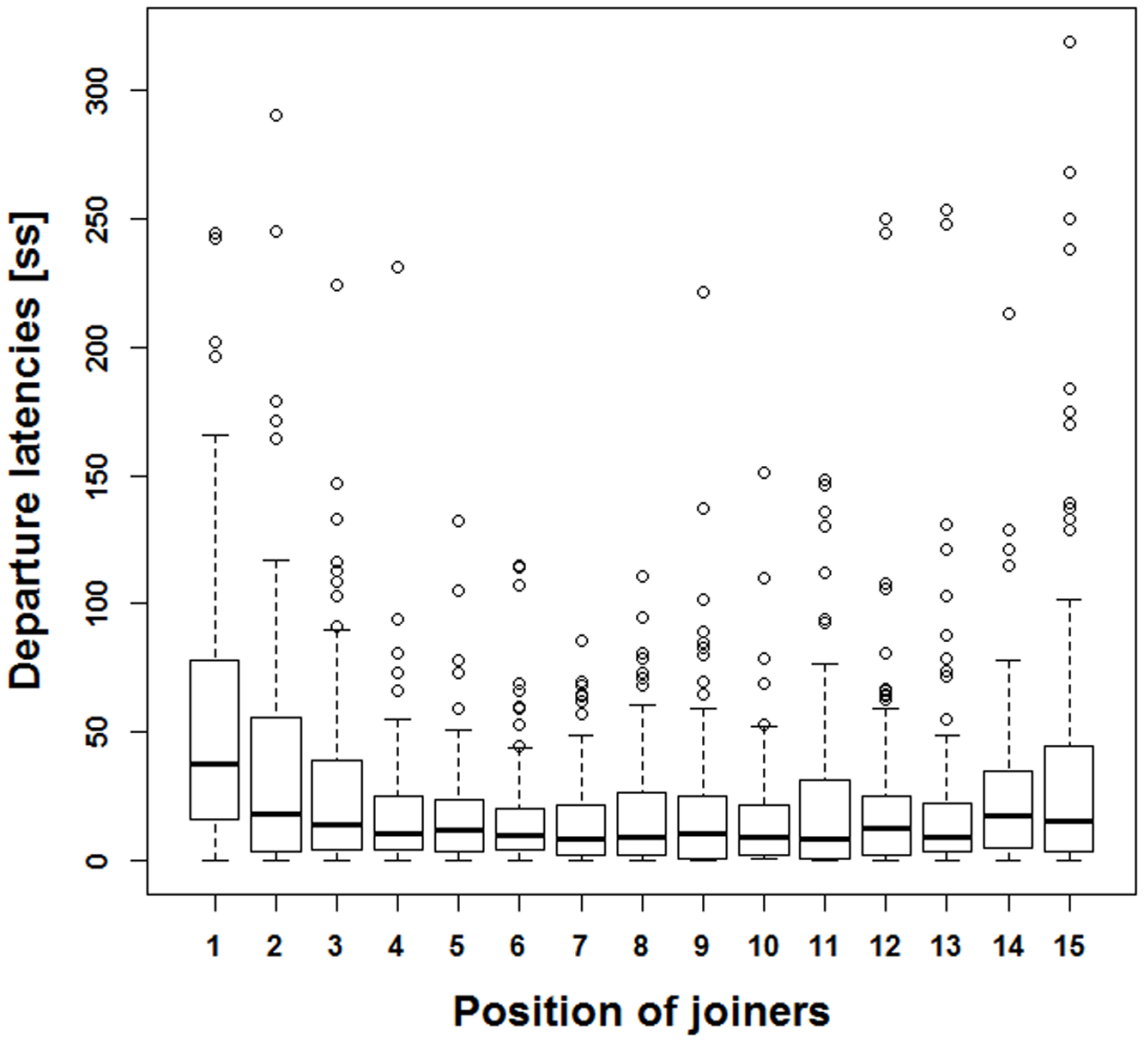
Departure latencies from the 1^st^ to 15^th^ joiner. The latencies of the first and second joiner were significantly longer than those from joiners 3 – 15 (Pairwise Wilcoxon rank sum tests: *P*<0.01).

**Figure 6:**
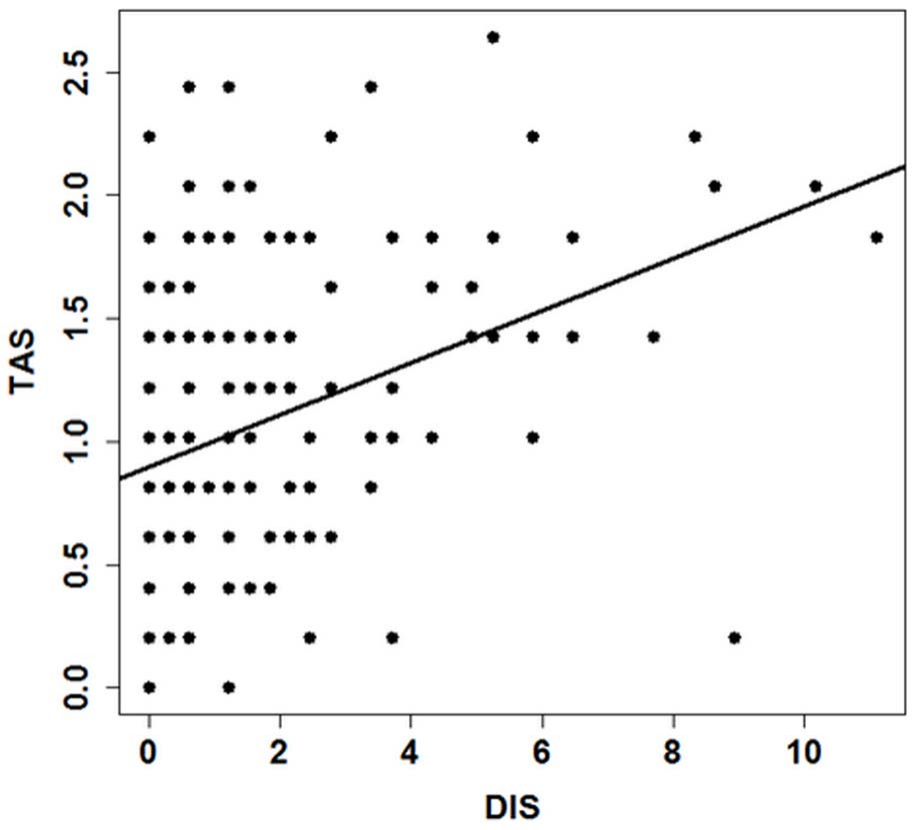
Relation between the dyadic association score and the travel association score during group movements. The strength of the social bond measured by the DIS (dyadic index of social relationships) and the TAS (travel association score) for each dyad were significantly positively correlated (Spearman rank correlation: *ρ* = 0.32, *N* = 276, *P*<0.001).

The distribution of departure latencies from the 1^st^ to the 15^th^ joiner was not uniform (Kruskal-Wallis test: *χ^2^* = 93.34, *df* = 14, *P*<0.001, Fig. 5). The latencies of the first and second joiner were significantly longer than those from joiners 3 – 15 (Pairwise Wilcoxon rank sum tests: P<0.01), suggesting that a mimetic mechanism determined the joining process. The average position in the progression order was independent of rank and CIS (Spearman rank correlation: rank: *ρ* = 0.34, *N* = 22, *P* = 0.12; CIS: *ρ* = –0.26, *N* = 24, *P* = 0.23). The strength of the social bond measured by the DIS and the travel association score for each dyad were significantly positively correlated (Spearman rank correlation: *ρ* = 0.32, *N* = 276, *P*<0.001, Fig. 6). Animals with a high DIS also had a high travel association score, or in other words: animals that were often found in body contact during the scans outside the movement context, travelled more often together than those found less often in body contact. Dyads with particularly high affiliation scores above 4 almost never had low travel association scores below 1.

## Discussion

We found that (i) individuals frequently showed pre-departure behaviour prior to collective movements, the initiator usually chose the direction for which more individuals voted and pre-departure behaviour was predominantly shown by adults. Further, (ii) higher ranking individuals frequently initiated collective movements and (iii) the joining process was determined by mimetism and affiliative relationships. Below, we discuss these findings in the context of other macaque studies (Table 2) focusing on (i) the pre-departure period in which individuals can show their preferred travel direction and timing, (ii) the initiation movement of an individual, choosing one specific time and direction for the departure and (iii) the joining of the movement by other group members.

**Table 2:** Comparative data of Barbary macaques (this study, wild group), rhesus macaques (semi-free ranging conditions), Japanese macaques (wild group) and Tonkean macaques (semi-free ranging conditions).

	Rhesus macaques	Japanese macaques	Barbary macaques	Tonkean macaques
**Grade of dominance style**	**1**	**1**	**3**	**4**
**Pre-departure behaviour**				
Occurrence before collective movements	9%^1^	not known	78%	29 –30%^1,2^
Display by the initiator before collective movements	5%^1^	not known	52%	23% – 57%^2^
Number of pre-departure displaying individuals	3.09±0.32^1^	not known	2.78±2.49	3.45±0.42^1^
Identity of pre-departure showing individuals	not specific^1^	not known	adults	not specific^1^
**Initiatorship**				
Age of frequent initiators	adults^1^	adults^3^	adults	not specific^1^
Sex of frequent initiators	not tested^1^	not specific^3^	males	not specific^1^
Rank of frequent initiators	not specific^1^	dominant^3^	dominant	not specific^1^
Social integration of frequent initiators	high^4^	not known	not specific/(low*)	high^4^
Effect of dominance on the success	positive^1^	not specific^3^	not specific	not specific^1^
Effect of social integration on the success	positive^4^	not known	not specific/(positive*)	positive^4^
**Joining of group movements**				
Influence of kinship	yes^5^	yes^3^(for females only)	not known	no^6^
Influence of affiliative relationships	not known	not known	yes	yes^6^
Individuals at the front of progression	dominant, males, socially integrated^5^	not known	not specific	not specific^5^
**Decision-making**				
Type of consensus decisions	partially shared^1^	partially shared^3^	partially shared	equally shared^1^
Influencing individuals	dominant adults, sex effect not known^1^	dominant adults, both sexes^3^	dominant males	-^1^

Except for Japanese macaques, study subjects were all group members, including juveniles. *only if including juveniles;^ 1^ Sueur and Petit 2008 [8]; ^2^ Sueur et al. 2010 [9]; ^3^ Jacobs et al. 2011a [12]; ^4^ Sueur et al. 2012 [58]; ^5^ Sueur and Petit 2008 [7]; ^6^ Sueur et al. 2009 [19].

### Pre-departure behaviour

The display of pre-departure behaviour in Barbary macaques is similar to that found in other macaque species [8], [9]. However, in this study an unusually high proportion of collective movements were preceded by pre-departure behaviour which may be due to the captive conditions of the Tonkean and rhesus macaque study [8], [9]. In an enclosure, possible directions to move are limited compared to those in the wild, and individuals are provided with sufficient food. Therefore, true conflicts of interests may be rare and communication of preferred travel timing and destination may not be as urgent as in natural conditions. In all studies a similar number of individuals (approximately 3, i.e. 14 – 39% of group members in Tonkean macaques, 13% of group members in Barbary macaques and 16% of group members in rhesus macaques, [8], [9]) were showing pre-departure behaviour before a collective movement. With regard to the proportion of collective movements involving pre-departure behaviour by the initiator itself Barbary macaques in this study were more similar to captive Tonkean than rhesus macaques.

Displaying pre-departure behaviour in more than one direction shows a conflict of interest between group members. If different directions were proposed during the pre-departure period, the one finally chosen by the initiator had more group members voting for this than the alternative. This suggests that the initiator compared the number of voting individuals and took the number of pre-departure displaying individuals into account when undertaking an initiation movement. The change in direction of displaying pre-departure behaviour indicates that the group members were able to assess the number of pre-departure displaying individuals for different directions and consequently achieve a consensus prior to departure of the initiator via a quorum threshold of three individuals. This small proportion of the group stresses the importance of the identity of the pre-departure displaying individuals. Adults played a prominent role before departure which appears plausible because of their greater experience and knowledge of the home range compared to juveniles [51], [16]. However, the small number of group members showing pre-departure behaviour is remarkable; a fourth of the adult individuals signaling in the same direction were enough to initiate a movement. This may represent a compromise between the risk of a wrong decision for the whole group due to a low proportion of voters and the time invested in the voting process, i.e. a speed-accuracy trade-off [18].

In contrast to this study where mainly adults displayed pre-departure behaviour, pre-departure behaviour was independent of age in Tonkean and rhesus macaques [8], [9]. Thus, Barbary macaques do not show intermediary behaviour between the intolerant and the tolerant macaque species. Our findings support the true signaling function of pre-departure behaviour. By negotiating over the travel direction the individuals can avoid a group fission and associated costs (e.g. reduced capacity to locate predators, like feral dogs, in a small group). The display of pre-departure behaviour is the easiest way to reach a consensus [25] and is much less costly than a group fission by mistake [17] which shows the adaptive value of pre-departure behaviour.

### Initiatorship

Every adult initiated a group movement, three juveniles never tried and the higher ranking an individual, the more often it tried to initiate collective movements. Interestingly, in the intolerant rhesus macaques, dominance rank does not influence the number of initiation movements, but age and social integration do: only adults initiate collective movements and social integration favors the initiation frequency. In the tolerant Tonkean macaques the initiation frequency was independent of sex, age and rank [8], [19], but positively affected by social integration as in rhesus macaques. In intolerant wild Japanese macaques adults and mainly high-ranking individuals initiate group movements. Thus, Barbary macaques seem to be more similar to intolerant Japanese macaques than intolerant rhesus and tolerant Tonkean macaques. The lacking dominance effect in rhesus macaques may result from captive conditions because dominance predicts initiation frequency in intolerant wild Japanese macaques.

The group members differed in their probability of successful initiations significantly only in the CIS and when including juveniles in the analyses. Animals with a higher CIS which were mostly juveniles had a higher percentage of successful initiation movements. But these successful movements were of shorter distance and rare, therefore high ranking adults had in total a stronger influence on the group’s movement. Again, these results most closely match those from intolerant Japanese macaque studied in the wild while in captivity rhesus macaque social integration also affected relative initiation success.

The fact that age is an important variable for the initiator’s identity was expected. Adults may have a better knowledge of the environment and therefore are more prominent to act as decision-makers [51]. The finding that successful initiation movements mainly were performed by high ranking group members may be explained by increased visual attention paid to higher ranking individuals ones than vice versa [52]. Subordinates may be aware of their low chances of initiation success and prefer to follow dominant individuals instead of initiating group movements themselves. In fact, dominants operate more autonomously and often possess a crucial role within the social network which allows them to influence group members more than others, maybe even passively [18]. Since dominant individuals were the main decision-makers in the study group in terms of initiation frequency, initiated longer group movements, and all males were dominant over females, males had more influence on the group decisions than females. Therefore, male dominance possibly outweighed the effect of superior ecological knowledge of philopatric females. Higher nutritional requirements of larger males have been suggested to explain their prominent role in group decision-making [27], [53], but females in our study were all either in the late stages of pregnancy or lactating which should render male and female energy requirements more similar.

In this study pre-departure behaviour occurred frequently and the role of the initiator was not restricted to a specific individual, suggesting that consensus decisions were not unshared. As adults had more influence in displaying pre-departure behaviour than juveniles and especially the high ranking males initiated collective movements the decision is reached neither by an entirely self-organized process nor by equally shared consensus. Instead, our results suggest that wild Barbary macaques use partially shared consensus decisions to coordinate group movements with the highest ranking adults acting as decision-making individuals. Contrary to our prediction, these were not individuals of a higher social integration.

### Joining process

The departure latencies were significantly higher for the first and second joiner and equal for the remainder of joiners suggesting that mimetism determines the joining process of a collective movement with a quorum threshold of two joining individuals. Joining order was neither significantly related to rank nor social integration, instead closely affiliated individuals travelled together adding a social constraint to the joining process. Thus, the decision to join a group movement depended not only on the number of pre-departure displaying individuals, but also on the behaviour of the individual’s most preferred affiliates and the number of individuals that had already joined. Among female macaques strong bonds are typically predicted by genetic relatedness [36], [12]. Male macaques, including Barbary macaques, have been shown to differentially affiliate with specific males and to rely on closely bonded partners as allies in agonistic coalitions [54], [55]. King et al. [56] also found that individuals follow “friends” in chacma baboons (*Papio ursinus*), but in this species dominant, male individuals travel at the front [57]. Contrasting results have been found for the effect of the CIS: individuals with a low as well as a high CIS have been found to travel at the front [31], [56].

Similar to this study, Tonkean macaques join group movements independently of rank but according to affiliative relationships [7], [19], [26]. Therefore, affiliative relationships seem to be an important driver of group cohesion. In macaque species with more pronounced kin preferences in social behavior and a strict hierarchical society, e.g. rhesus and Japanese macaques, the joining order is constrained by kinship [7], [12], dominance [7] and social integration [7]. In Tonkean macaques kin preferences are less pronounced which may explain the different social characteristics influencing the joining process. In this study information on kinship was lacking, but the fact that affiliative relationships significantly influenced the joining process may indicate that Barbary macaques behaved more similar to tolerant than extremely intolerant macaques in terms of factors driving the joining process.

The different influence of the hierarchical rank order among the macaque species may reflect a stepwise relationship: only in extremely intolerant species (and possibly some intermediary species) dominant individuals travelled at the front whereas in other intermediary and the most tolerant species the effect is totally absent.

### Influence of social style on organization of group coordination in macaques

Partially shared consensus decisions mediated by selective mimetism based on affiliative relationships seemed to be prevalent in this study group, largely supporting our predictions. Initiators gave up and turned around when no group members followed (an event that accounted for less than 3% of initiation movements), displayed pre-departure behaviour in more than half of cases prior to group movements and usually chose the direction in which more group members displayed pre-departure behaviour. This shows that the initiators reacted to the behaviour of their conspecifics and aimed for a consensus [11]. Pre-departure displaying individuals also changed the direction of their behaviour indicating that they also reacted to the behaviour of other group members. The same phenomenon was observed in Tonkean macaques [9], [27], [26].

In Tonkean macaques equally shared consensus decisions were prevalent, probably due to their shallow dominance gradient and pronounced social tolerance [8]. This shows that Barbary macaques are in one aspect of group coordination, the type of decision-making, more similar to rhesus and Japanese than Tonkean macaques, but in terms of another aspect, the joining process, their relative position could not be determined. They may be more similar to Tonkean macaques because in both species the joining order was independent of rank but influenced by affiliative relationships [19], [26], but unfortunately it could not be tested whether kinship would have provided a better explanation for the progression order. The relationship between social tolerance and decision-making does not seem to be a continuous one. Only the most tolerant species seem to use equally shared consensus decisions while the extremely intolerant as well as the intermediary species use partially shared consensus decisions.

Overall, our results support the hypothesis that collective movements are influenced by the social structure in macaques, but it has to be distinguished between the single characteristics, i.e. joining order and type of decision-making. Stepwise relationships were found for both, but in different directions. Since only four of the more than 20 macaque species have been studied so far, further work has to be done on wild populations of more, especially intermediate species, to confirm the relation between social style and processes of group coordination in macaques.

## Supporting Information

Figure S1 Results of the pilot study. Pyritz et al.’s [14] procedure was followed to generate operational definitions for movement related terms. Every group member, except one 1 year old juvenile that was limping during the period of the pilot study, was observed for 20 minutes using focal animal sampling in six different time slots equally distributed over the day yielding frequencies of (A) latencies between two movements (*N* = 1870), (B) the covered distances during movements (*N*  =  1327) and (C) distances to the nearest neighbour after movements (*N*  =  1604). Arrows indicate the estimated thresholds for operational group movement definitions derived from the frequency distribution (A: 3.5 minutes, B: 18 meters, C: 11 meters). To improve clarity, representation latencies of less than 0.5 (*N*  =  1328) and more than 6 minutes (*N* = 14) are not depicted in A.
(TIF)
