# Supplementary figures and images for: The Organization of Collective Group Movements in Wild Barbary Macaques (Macaca sylvanus): Social Structure Drives Processes of Group Coordination in Macaques
Source: PLoS One. 2013 Jun 21;8(6):e67285. doi: 10.1371/journal.pone.0067285 (PMC3689719; doi:10.1371/journal.pone.0067285)

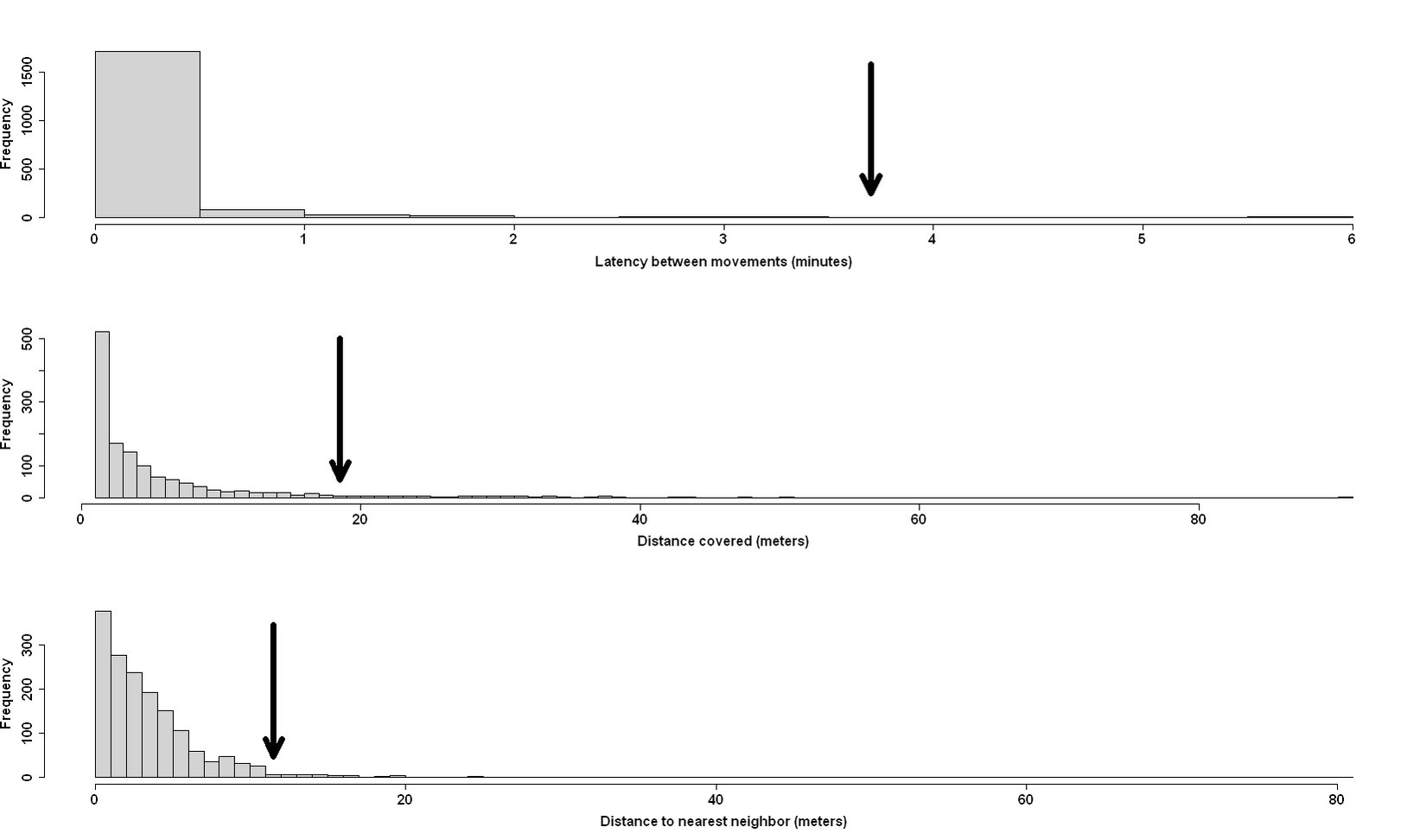

Supplement: Figure S1 — Results of the pilot study. Pyritz et al.’s [14] procedure was followed to generate operational definitions for movement related terms. Every group member, except one 1 year old juvenile that was limping during the period of the pilot study, was observed for 20 minutes using focal animal sampling in six different time slots equally distributed over the day yielding frequencies of (A) latencies between two movements (N = 1870), (B) the covered distances during movements (N = 1327) and (C) distances to the nearest neighbour after movements (N = 1604). Arrows indicate the estimated thresholds for operational group movement definitions derived from the frequency distribution (A: 3.5 minutes, B: 18 meters, C: 11 meters). To improve clarity, representation latencies of less than 0.5 (N = 1328) and more than 6 minutes (N = 14) are not depicted in A. (TIF) [file pone.0067285.s001.tif]
